# Baseline comorbidity of cardiovascular-kidney-metabolic syndrome increases the risk of adverse clinical outcomes in patients with chronic kidney disease

**DOI:** 10.3389/fendo.2025.1563164

**Published:** 2025-04-16

**Authors:** Jiali Meng, Wen Li, Wenjing Fu, Aihua Zhang

**Affiliations:** ^1^ Department of Nephrology, Xuanwu Hospital, Capital Medical University, Beijing, China; ^2^ The National Clinical Research Center for Geriatric Disease, Xuanwu Hospital, Capital Medical University, Beijing, China

**Keywords:** cardiovascular-kidney-metabolic syndrome (CKM), chronic kidney disease, all-caused mortality, end-stage renal disease (ESRD), metabolic disease (MD)

## Abstract

**Introduction:**

Our study aims to analyze the relationship between different stage of Cardiovascular-Kidney-Metabolic (CKM) Syndrome in Chronic Kidney Disease (CKD) patients and the risk of progression to all-caused mortality or end-stage renal disease (ESRD).

**Methods and results:**

A retrospective cohort study was performed by collecting baseline data of CKD patients. All participants were followed throughout the course of the study. Cox proportional hazards analysis and Fine-Gray subdistribution model was performed to analyze the prognostic value of different CKM stages on the risk of adverse clinical outcomes (all-caused mortality or progression to ESRD) of these patients. 1,358 patients finally completed the follow-up. Among them, 1,233 patients were alive, and 125 patients had died; and 163 patients progressed to ESRD. Baseline CKM stage 3 (OR=3.906, 95% CI=0.988-16.320, p=0.048) and stage 4 (OR=5.728, 95% CI=1.329-24.698, p=0.019) remain independent risk factors for all-cause mortality in CKD patients, while CKM stage 2b (OR=2.739, 95% CI=1.157-6.486, p=0.022) were identified as having an independent risk factor for progression to ESRD in CKD patients by adjusting confounding factors.

**Conclusion:**

Our research demonstrated that a high-risk CKM stage can predict adverse clinical outcomes in CKD patients, including all-cause mortality and progression to ESRD.

## 1 Introduction

Cardiovascular-Kidney-Metabolic Syndrome (CKM) is a new concept proposed by the American Heart Association (AHA), which is defined as a systemic disease resulting from the pathophysiological interactions between chronic kidney disease (CKD), cardiovascular disease (CVD), and cardiometabolic risk factors such as dyslipidemia, hypertension, obesity, insulin resistance, and hyperglycemia (1). The pathophysiological interactions between metabolic risk factors such as tissue fat accumulation, diabetes, hypertension, hyperlipidemia, CKD, and the cardiovascular system led to multi-organ dysfunction and a high incidence of adverse cardiovascular outcomes (2). There have been studies on the impact of cardiovascular-kidney-metabolic syndrome on the mortality risk of in subgroup CKD patients as well as general population (3) but there is no systematic research on the effects of different risk stage of CKM on the progression of CKD patients to different outcomes. Our study aims to analyze the relationship between CKM stage in CKD patients and the risk of progression to all-caused mortality or end-stage renal disease (ESRD).

## 2 Method

### 2.1 Data source and study population

**Figure 1:**
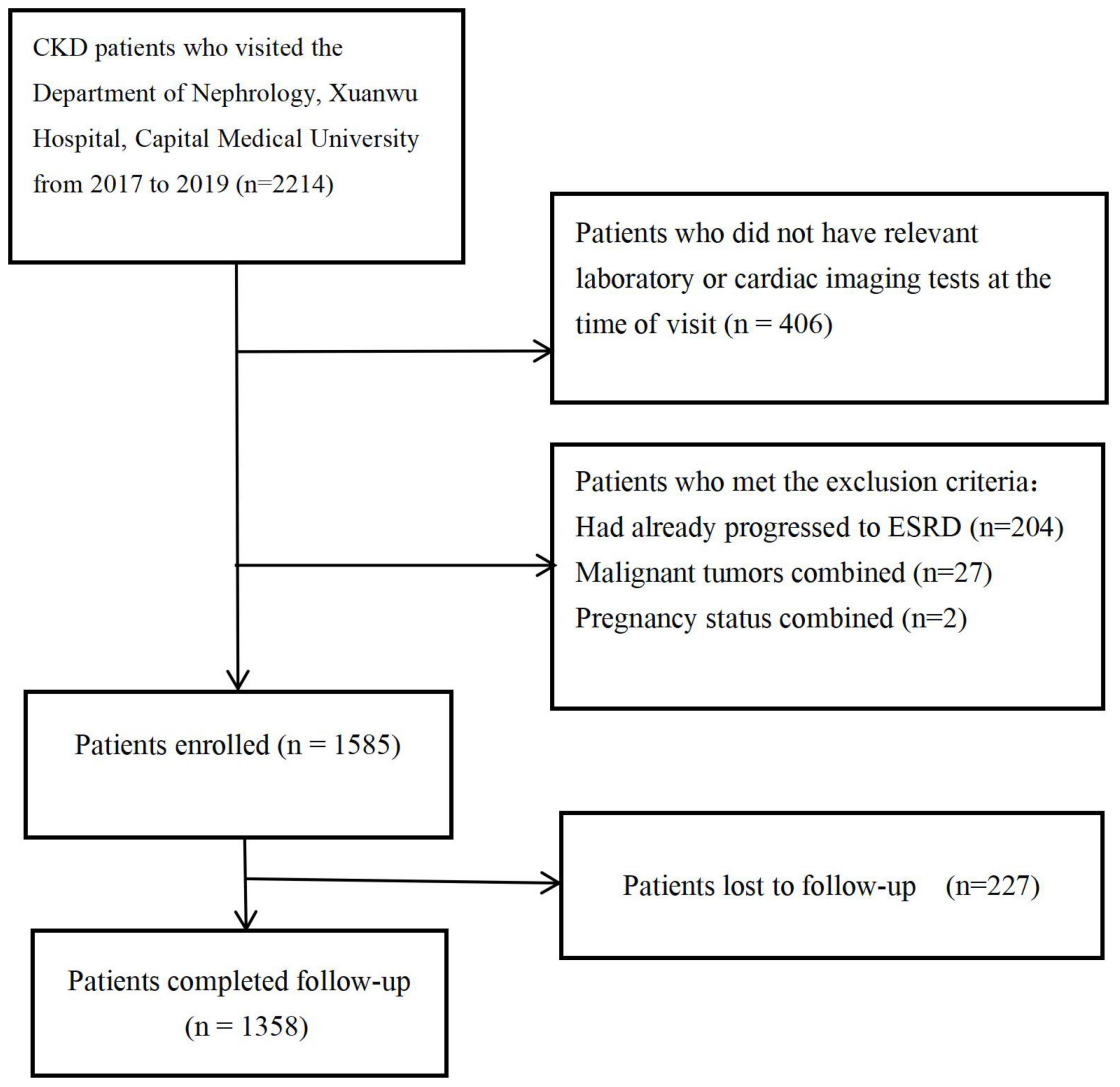
The inclusion criteria and study process for patients in this study.

This study is a single-center cohort study. We collected data from 2,215 patients who visited the Nephrology Department of Xuanwu Hospital, Capital Medical University, from December 2017 to December 2019. The Ethics Committee of Xuanwu Hospital, Capital Medical University, approved this study (2019-130). Since this is a retrospective study, the requirement for informed consent was waived. Patients who met the definition of chronic kidney disease according to the KDIGO 2024 guideline (an eGFR of less than 60 mL/min/1.73 m^2^ for more than three months, and/or clinical evidence of kidney damage) (4), and who underwent routine blood tests, liver and kidney function tests, serum electrolyte tests, urine protein quantification, laboratory tests, and coronary CTA or echocardiography, and agreed to regular follow-up via telephone or outpatient visits were included. Patients who had already progressed to ESRD (eGFR < 15 mL/min/1.73 m^2^) (4), have malignant tumors or pregnancy at the time of initial diagnosis were excluded. A total of 1,585 patients with chronic kidney disease were included in the cohort, with follow-up ending in January 2023, 227 patients lost follow-up. The inclusion criteria and study process for patients are shown in **Figure 1**.

### 2.2 Data collection

General information was collected from patients at the time of their initial diagnosis, including gender, age, weight, body mass index (BMI), and metabolic comorbidities (hypertension, diabetes, hyperlipidemia). Blood test results were also collected from fasting samples taken at the time of initial diagnosis, including hemoglobin, white blood cells, platelets, albumin, lipid profile, uric acid, and electrolytes (calcium, phosphorus), among others. Additionally, cardiac ultrasound or coronary CTA imaging data were collected. All imaging examinations were conducted and measured by experienced radiologists, who also provided the reports.

#### 2.2.1 Definition of CKM stage

The stage of CKM syndrome was defined into 5 groups at the time of patients’ initial visit, which was based on the standards recommended by the AHA (4), taking into account the patient’s CKD stage, comorbidities, BMI, laboratory results, and imaging findings. Patients without any clinical or potential cardiovascular, renal, or metabolic risk factors are classified as stage 0, those with potential metabolic risks are stage 1, with clinical renal and metabolic abnormalities are stage 2, with subclinical cardiovascular disease or classified as high-risk CKD patients are stage 4, and stage 5 patients have already progressed to clinical ASCVD or heart failure. Because all enrolled patients had CKD, there was no CKM stage 0 or 1 in this study. Additionally, CKM stage 2 was divided into two sub-groups(CKM 2a and CKM 2b), in order to distinguish whether CKD patients with CKM stage 2 had concurrent metabolic disease or not. The details of the risk grading criteria in this study is shown in **Table 1**.

**Table 1:** The risk grading criteria for cardiovascular-kidney-metabolic (CKM) syndrome.

CKM stage	Grading standards
0	Without any metabolic risk factors or CKD
1	With only visceral fat accumulation
2a	CKD without metabolic risk factors or with only visceral fat accumulation
2b	CKD combined with metabolic diseases (hypertension/T2D/hyperlipidemia/overweight)
3	CKD combined with subclinical atherosclerotic cardiovascular disease (ASCVD) detected by coronary CTA And/or subclinical heart failure (HF) detected by echocardiography And/or CKD classified as high-risk by the KDIGO guidelines (3)
4	CKD combined with clinically confirmed cardiovascular disease (coronary heart disease/heart failure/atrial fibrillation, etc.)

CKD, chronic kidney disease; CKM, cardiovascular-kidney-metabolic syndrome; T2D, type 2 diabetes; CTA, Computed Tomography Angiography.

#### 2.2.2 CVD diagnosis

All CVD cases were diagnosed using patient clinical or imaging data. Clinically confirmed CVD was defined as having been diagnosed as CVD (heart attack, coronary heart disease, angina, congestive heart failure, or other heart problems) by a doctor during the patient’s medical history. Subclinical atherosclerotic cardiovascular disease (ASCVD) was defined as coronary artery calcification of grade II or higher (Agatston calcium score ≥ 1) (5). Subclinical heart failure(HF) was indicated by echocardiograph evidence of ventricular systolic or diastolic dysfunction, but without clinical symptoms or signs of heart failure.

#### 2.2.3 Etiology and risk classification of CKD

The etiological classification of chronic kidney disease was divided into five types: diabetic nephropathy, hypertensive nephropathy, Glomerulonephritis, polycystic kidney disease and other recorded diagnoses (drug-induced kidney damage, nephrotic syndrome, and so on). The glomerular filtration rate (GFR) was estimated using the CKD-EPI formula recommended by KDIGO 2024 for eGFR calculation and was stratified according to the CKD staging and grading criteria proposed by KDIGO 2024 guideline (4).

### 2.3 Follow-up

Patients were followed up by telephone or outpatient visits starting 3-6 months after diagnosis, continuing until the patient’s death, progression to ESRD, or loss to follow-up. The criteria for ESRD were defined as the initiation of renal replacement therapy (hemodialysis, peritoneal dialysis, or kidney transplantation), or an eGFR of less than 15 mL/min/1.73 m^2^).Information was recorded regarding the patient’s survival status, the time of death or progression to ESRD, causes of death, and reasons for loss to follow-up. The follow-up period ended in January 2023.

### 2.4 Statistical analysis

Results are expressed as proportions (percentages) for categorical variables, means ± standard deviation (S.D.) for continuous normally distributed variables, and medians (interquartile) for continuous non-normally distributed variables. The Student’s t-test was employed to compare differences between the two groups for normally distributed data, while the Mann-Whitney U test was used for non-normally distributed data. The counting data was expressed by percentage or composition ratio, and the comparison between groups was expressed by X^2^ test. Univariate Cox regression analysis is employed to determine whether the baseline CKM stage is a risk factor for all-cause mortality or progression to ESRD. Additionally, other potential risk factors for death or ESRD are identified from the baseline data and included in a multivariate Cox regression analysis to compare their correlation with the incidence of adverse outcomes. As all-cause mortality was considered as a competing risk to ESRD in our study design, the Fine-Gray subdistribution hazard model was employed to evaluate the association between CKM stages and adverse outcomes while accounting for competing events, in order to fit the real clinical scenarios. To ensure consistency in the analysis results, in the Fine-Gray model and Cox regression analyses before and after including other potential risk factors, patients in other CKM stages are compared with those in the CKM2a category. All statistical analyses are conducted using R (version 4.4.1) and SPSS 34.0, and the CBCgrps package is used to analyze baseline characteristics and create baseline data tables (6). Differences are considered statistically significant at P < 0.05.

## 3 Results

This study included a total of 1,585 patients with chronic kidney disease. By the end of the follow-up, 1,358 patients completed the follow-up, while 227 patients were lost to follow-up. The median follow-up time was 5.03 years. Among them, 1,233 patients were alive, and 125 patients had died; and 163 patients progressed to ESRD. The causes of death were as follows: cardiovascular disease in 51 cases, cerebrovascular disease in 12 cases, infections in 14 cases, multi-organ failure in 19 cases, gastrointestinal bleeding in 3 cases, and other or unknown causes in 26 cases. The baseline characteristics of patients were categorized based on whether they experienced all-cause mortality and whether they progressed to ESRD is shown in **Table 2**.

**Table 2:** Baseline characteristics of patients stratified by all-cause mortality and progression to ESRD.

Items	Conclusion (n=1358)	All-Cause mortality				Progression to ESRD			
		Non-death (n=1233)	Death (n=125)	t/X2	P	Non-ESRD (n=1195)	ESRD (n=163)	t/X2	P
CKM [n (%)]				67.978	<.001			22.497	<.001
2a	145	143 (11.6)	2 (1.6)			139 (11.6)	6 (3.7)		
2b	459	444 (36.0)	15 (12.0)			418 (35.0)	41 (25.2)		
3	432	385 (31.2)	47 (37.6)			359 (30.0)	73 (44.8)		
4	322	261 (21.2)	61 (48.8)			279 (23.3)	43 (26.4)		
Gender [male, n (%)]		634 (51.4)	69 (55.2)	0.65	0.42				
Age (Y)	58.50 ± 17.01	57.11 ± 16.95	72.27 ± 10.10	96.489	<.001	58.69 ± 17.22	57.10 ± 15.32	1.252	0.263
Cause of CKD [n (%)]				33.85	<.001			6.92	0.140
Glomerulonephritis	394	377 (30.6)	17 (13.6)			353 (29.5)	41 (25.2)		
Hypertensive nephropathy	384	342 (27.7)	42 (33.6)			338 (28.3)	46 (28.2)		
Diabetic Nephropathy	227	187 (15.2)	40 (32.0)			200 (16.7)	27 (16.6)		
polycystic kidney	16	14 (1.1)	2 (1.6)			11 (0.9)	5 (3.1)		
Other causes	337	313 (25.4)	24 (19.2)			293 (24.5)	44 (27.0)		
With metabolic comorbidities [n (%)]									
hypertension	1052	939 (76.2)	113 (90.4)	13.192	<.001	917 (76.7)	135 (82.8)	3.043	0.081
T2D	467	401 (32.5)	66 (52.8)	20.683	<.001	404 (33.8)	63 (38.7)	1.491	0.222
With ASCVD [n (%)]	322	261 (21.2)	61 (48.8)	47.906	<.001	279 (23.7)	43 (26.4)	0.729	0.393
With subclinical heart disease [n (%)]	444	369 (29.9)	75 (60.0)	22.553	<.001	404 (33.8)	40 (24.5)	0.329	0.566
eGFR (ml/min/1.73m^2^)	54.80 ± 27.21	56.09 ± 27.74	42.05 ± 16.78	30.883	<.001	56.28 ± 26.53	56.27 ± 29.68	29.887	<.001
Creatinine (umol/L)	134.67 ± 59.39	132.75 ± 59.17	153.66 ± 58.47	14.216	<.001	129.48 ± 55.16	172.78 ± 73.88	80.762	<.001
Hb (g/L)	123.87 ± 22.72	124.98 ± 22.57	113.15 ± 21.47	23.863	<.001	125.11 ± 22.62	115.41 ± 21.70	21.099	<.001
WBC (10^9/L)	6.99 ± 2.40	7.02 ± 2.37	6.80 ± 2.67	0.694	0.405	6.98 ± 2.413	7.124 ± 2.324	0.405	0.525
PLT (10^9/L)	213.23 ± 66.28	213.51 ± 64.91	210.13 ± 79.78	0.205	0.651	211.88 ± 66.54	222.48 ± 63.89	2.842	0.092
24-hour urine protein measurement (g/24h)	0.360 ± 0.919	0.180 ± 0.847	0.379 ± 0.924	3.402	0.065	0.358 ± 0.903	0.377 ± 1.04	0.035	0.851
Calcium (mg/dl)	8.88 ± 1.00	8.90 ± 1.03	8.66 ± 0.670	5.332	0.021	8.896 ± 0.994	8.77 ± 1.051	2.031	0.154
Phosphorus (mg/dl)	3.75 ± 0.767	3.75 ± 0.777	3.80 ± 0.650	0.342	0.559	3.738 ± 0.751	3.906 ± 0.868	6.187	0.013
Ca*P (mg^2^/dl^2^)	33.56 ± 6.71	33.62 ± 6.75	32.93 ± 6.09	0.978	0.323	33.41 ± 6.596	34.70 ± 7.398	4.656	0.031
Albumin (g/L)	38.07 ± 7.81	38.32 ± 8.19	35.08 ± 7.73	13.006	<.001	68.87 ± 7.789	36.87 ± 7.937	3.458	0.063
ALT(U/L)	21.65 ± 37.11	22.11 ± 38.61	16.55 ± 8.92	1.862	0.173	21.82 ± 39.13	20.42 ± 16.27	0.169	0.681
LDL-C (mmol/L)	3.12 ± 1.45	3.12 ± 1.48	3.07 ± 1.21	0.112	0.738	3.107 ± 1.394	3.252 ± 1.855	1.17	0.28

CKD, chronic kidney disease; CKM, cardiovascular-kidney-metabolic syndrome; T2D, type 2 diabetes; ASCVD, atherosclerotic cardiovascular disease; Hb, hemoglobin; WBC, white blood cells; PLT, platelets; ALT, alanine aminotransferase; LDL-C, Low-Density Lipoprotein Cholesterol; eGFR, estimated glomerular filtration rate.

### 3.1 Comparison of all-cause mortality differences among CKD patients with different CKM stage

**Figure 2:**
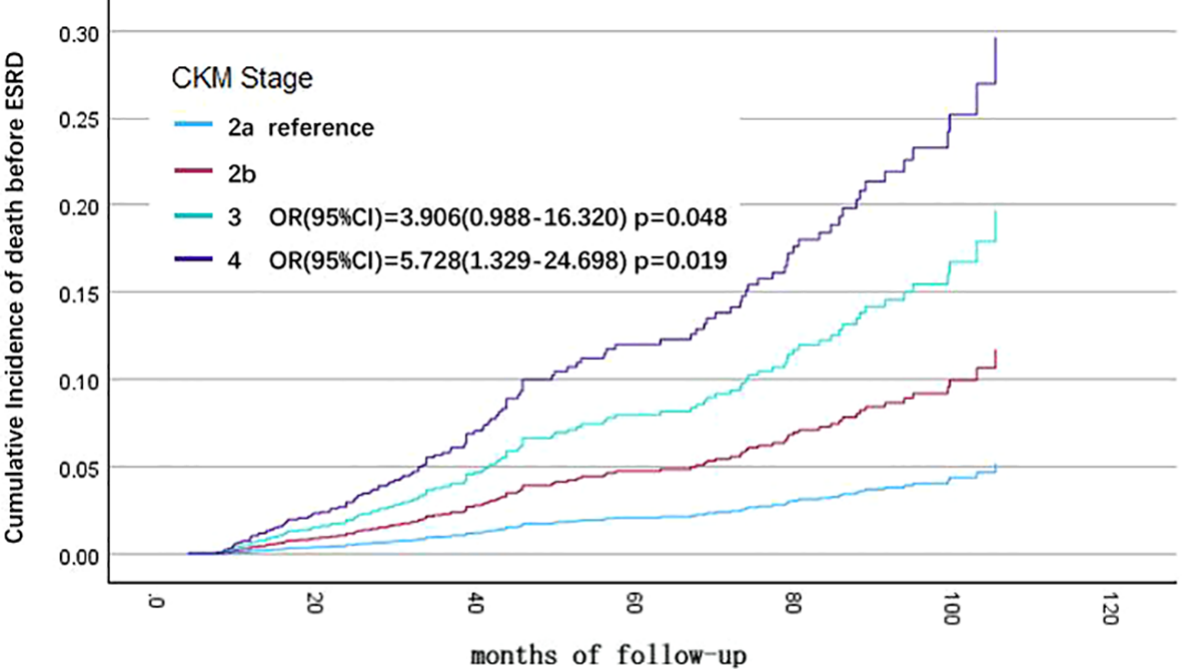
Cumulative incidence curves for end-stage renal disease (ESRD) by cause of different cardiovascular-kidney-metabolic (CKM) syndrome stages (by multivariate COX regression analysis). The cumulative risk has been adjusted for the effect of eGFR, hemoglobin, albumin, LDL-C, and cause of CKD.

Univariate Cox regression analysis showed that different CKM stage, age, eGFR, hemoglobin, serum albumin, low-density lipoprotein, and cause of CKD were associated with all-cause mortality in CKD patients. Variables with P < 0.1 from the univariate Cox regression analysis were included in the multivariate Cox regression analysis for statistically significant risk factors (**Table 3**). The cumulative risk curves for all-cause mortality in CKD patients with different baseline CKM risk stage over time are shown in **Figure 2**. The results indicate that, after excluding the influence of other related risk factors, baseline CKM stage 3 (OR=3.906, 95% CI=0.988-16.320, p=0.048) and stage 4 (OR=5.728, 95% CI=1.329-24.698, p=0.019) remain independent risk factors for all-cause mortality in CKD patients.

**Table 3:** Analysis of risk factors associated with All-Cause mortality before progress to end-stage renal disease (ESRD) in CKD patients (univariate and multivariate COX regression analysis).

Items	Univariate COX regression		Multivariate COX regression	
	HR (95%CI)	p value	HR (95%CI)	p value
CKM stage				
2a	reference		reference	
2b	4.388 (1.025-18.786)	0.046	2.267 (0.514-9.994)	0.281
3	7.096 (1.704-29.549)	0.003	3.906 (0.988-16.320)	0.048
4	16.463 (4.018-67.446)	<0.001	5.728 (1.329-24.698)	0.019
Gender				
female	reference			
male	1.015 (0.701-1.471)	0.935		
Age (Y)				
<60	reference		reference	
≥60	5.330 (3.906-9.176)	<0.001	2.599 (1.525-4.430)	<0.001
Cause of CKD				
Glomerulonephritis	reference		reference	
Hypertensive nephropathy	5.727 (2.665-12.308)	<0.001	1.351 (0.729-2.504)	0.34
Diabetic Nephropathy	15.578 (7.284-33.314)	<0.001	2.326 (1.265-4.279)	0.007
polycystic kidney	14.988 (3.954-56.81)	<0.001	1.580 (0.352-7.103)	0.551
Other Recorded Diagnoses	4.77 (2.141-10.625)	<0.001	1.033 (0.542-1.968)	0.923
eGFR (ml/min/1.73m^2^)				
≥60	reference		reference	
30-60	2.785 (1.639-4.734)	<0.001	1.247 (0.713-2.181)	0.44
<30	4.033 (2.193-7.416)	<0.001	1.153 (0.594-2.238)	0.674
Hb (g/L)				
<110	3.869 (2.596-5.766)	<0.001	2.032 (1.291-3.199)	0.002
≥110	reference		reference	
WBC (10^9/L)	0.955 (0.870-1.049)	0.334		
PLT (10^9/L)	0.999 (0.995-1.003)	0.543		
Albumin (g/L)				
<30	3.488 (2.231-5.454)	<0.001	3.341 (2.082-5.361)	<0.001
≥30	reference		reference	
Ca*P (mg^2^/dl^2^)				
<30	1.577 (0.806-2.390)	0.157		
30-39	reference			
≥40	1.309 (0.725-2.364)	0.371		
24-hour urine protein measurement (g/24h)				
<0.15	reference			
≥0.15	0.544 (0.122-1.527)	0.341		
LDL-C (mmol/L)				
<1.8	reference		reference	
1.8-2.6	0.531 (0.283-0.999)	0.061	0.84 (0.442-1.597)	0.594
≥2.6	0.496 (0.248-0.978)	0.050	0.702 (0.370-1.334)	0.28
ALT(U/L)				
<40	reference			
≥40	0.142 (0.020-1.215)	0.152		

CKD, chronic kidney disease; CKM, cardiovascular-kidney-metabolic syndrome; Hb, hemoglobin; WBC, white blood cells; PLT, platelets; ALT, alanine aminotransferase; LDL-C, Low-Density Lipoprotein Cholesterol; eGFR, estimated glomerular filtration rate.

Additionally, baseline age > 60 years (OR=2.599, 95% CI=1.525-4.430, p<0.001), primary disease being diabetic nephropathy (compared to glomerulonephritis, OR=2.326, 95% CI=1.265-4.279, p=0.007), hemoglobin < 110 g/L (OR=2.032, 95% CI=1.291-3.199, p=0.002), and serum albumin < 30 g/L (OR=3.341, 95% CI=2.082-5.361, p<0.001) are also independent risk factors for all-cause mortality in CKD patients by multivariate Cox regression analysis.

### 3.2 Comparison of differences in progression to ESRD among CKD patients with different CKM stage

**Figure 3:**
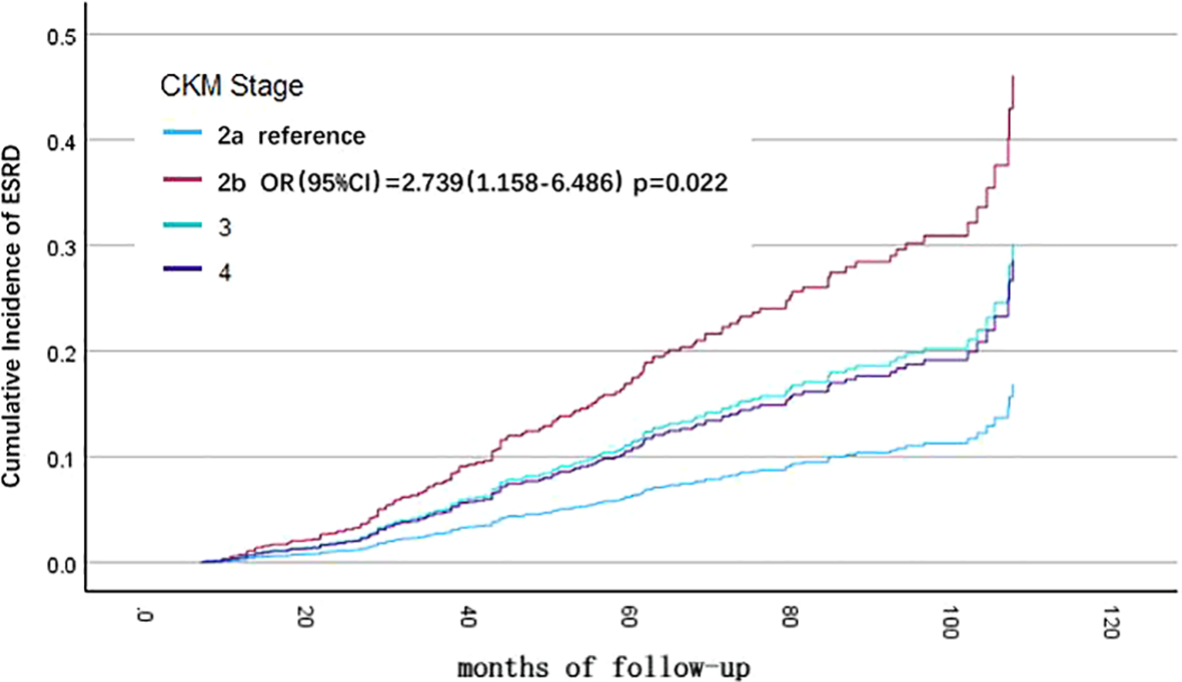
Cumulative incidence curves for death from any cause before progression to end-stage renal disease (ESRD), by cause of different cardiovascular-kidney-metabolic (CKM) syndrome stages (by multivariate COX regression analysis). The cumulative risk has been adjusted for the effect of cause of CKD, baseline eGFR level, hemoglobin, calcium-phosphorus product, albumin, and LDL-C.

Univariate Cox regression analysis showed that CKM stage, cause of CKD, baseline eGFR level, hemoglobin, calcium-phosphorus product, albumin, and low-density lipoprotein were associated with progressing to ESRD in CKD patients. Variables with P < 0.1 from the univariate Cox regression analysis were included in the multivariate Cox regression analysis for statistically significant risk factors (**Table 4**). The cumulative risk curves for CKD patients progressing to ESRD with different baseline CKM stage over time are shown in **Figure 3**. Compared to patients with CKM stage 2a, those with CKM stage 2b (OR=2.739, 95% CI=1.157-6.486, p=0.022) were identified as having an independent risk factor for progression to ESRD in CKD patients.

**Table 4:** Analysis of risk factors associated with end-stage renal disease (ESRD) in CKD patients (univariate and multivariate COX regression analysis).

Items	Univariate COX regression		Multivariate COX regression	
	HR (95%CI)	p value	HR (95%CI)	p value
CKM stage				
2a	reference		reference	
2b	3.06 (1.298-7.211)	0.011	2.739 (1.157-6.486)	0.022
3	5.259 (2.287-12.095)	<0.001	1.794 (0.723-4.447)	0.207
4	4.113 (1.75-9.669)	0.001	1.699 (0.673-4.292)	0.262
Gender				
woman	reference			
man	0.744 (0.547-1.311)	0.259		
age (Y)				
<60	reference			
≥60	0.785 (0.577-1.068)	0.123		
Cause of CKD				
glomerular nephritis	reference		reference	
Hypertensive nephropathy	1.413 (0.926-2.156)	0.109	1.023 (0.639-1.640)	0.923
diabetic nephropathy	1.981 (1.213-3.235)	0.006	1.044 (0.602-1.812)	0.877
polycystic kidney	4.638 (1.824-11.792)	0.001	2.647 (0.996-7.038)	0.049
Other causes	1.717 (1.121-2.629)	0.013	1.056 (0.669-1.669)	0.814
eGFR (ml/min/1.73m^2^)				
≥60	reference		reference	
30-60	1.268 (0.825-1.949)	0.278	1.480 (0.881-2.488)	0.139
<30	5.686 (3.738-8.650)	<0.001	5.487 (2.946-10.218)	<0.001
Hb (g/L)				
<110	3.944 (2.824-5.506)	<0.001	1.904 (1.279-2.836)	0.002
≥110	reference		reference	
WBC (10^9/L)	1.016 (0.948-1.088)	0.651		
PLT (10^9/L)	1.002 (1.000-1.005)	0.116		
Albumin (g/L)				
<30	2.437 (1.604-3.701)	<0.001	1.919 (1.220-3.019)	0.005
≥30	reference		reference	
Ca*P (mg^2^/dl^2^)				
<30	0.992 (0.703-1.402)	0.965	1.143 (0.803-1.629)	0.458
30-39	reference		reference	
≥40	1.920 (1.259-2.928)	0.002	1.308 (0.848-2.018)	0.225
24-hour urine protein measurement (g/24h)				
<0.15	reference			
≥0.15	0.653 (0.402-1.060)	0.105		
LDL-C (mmol/L)				
<1.8	reference		reference	
1.8-2.6	0.464 (0.263-0.820)	0.008	0.519 (0.291-0.928)	0.027
≥2.6	0.626 (0.361-1.086)	0.096	0.743 (0.425-1.300)	0.298
ALT(U/L)				
<40	reference			
≥40	0.938 (0.479-1.837)	0.852		

CKD, chronic kidney disease; CKM, cardiovascular-kidney-metabolic syndrome; Hb, hemoglobin; WBC, white blood cells; PLT, platelets; ALT, alanine aminotransferase; LDL-C, Low-Density Lipoprotein Cholesterol; eGFR, estimated glomerular filtration rate.

Moreover, CKD caused by polycystic kidney (compared to glomerulonephritis, OR=2.647, 95% CI=0.996-7.038, p=0.049), eGFR < 30 ml/min/1.73 m^2 (OR=5.487, 95% CI=2.946-10.218, p<0.001), baseline anemia (OR=1.904, 95% CI=1.279-2.836, p=0.002), and albumin < 30 g/L (OR=1.919, 95% CI=1.220-3.019, p=0.005) were also identified as risk factors for progression to ESRD in CKD patients. In contrast, baseline serum low-density lipoprotein levels between 1.8-2.6 mmol/L (OR=0.519, 95% CI=0.291-0.928, p=0.027) were found to be protective factors for renal function in CKD patients by multivariate Cox regression analysis.

### 3.3 Competing risks analysis of ESRD progression and all-cause mortality in CKD patients

**Figure 4:**
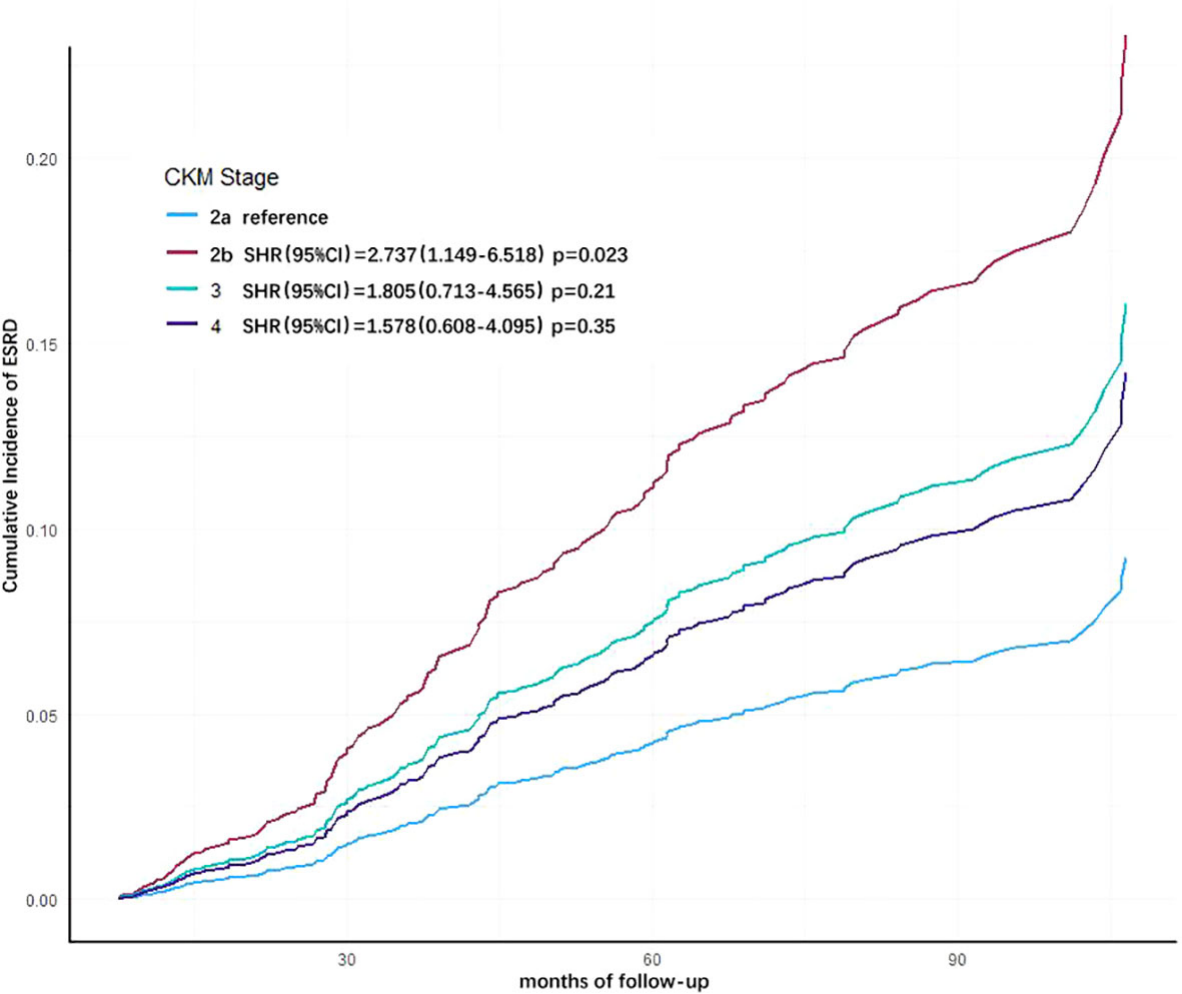
Cumulative incidence curves for end-stage renal disease (ESRD) by cause of different cardiovascular-kidney-metabolic (CKM) syndrome stages (by Fine-Gray subdistribution hazard model). The cumulative risk has been adjusted for the competitive risk of all-cause mortality, as well as the effect of age, eGFR, hemoglobin, albumin, LDL-C, and cause of CKD.

The Fine-Gray sub-distribution hazard model was employed to account for the competing risk of all-cause mortality when analyzing progression to ESRD. Consistent with the multivariate COX regression analysis, covariates including etiology of CKD, baseline eGFR level, hemoglobin, calcium-phosphorus product, albumin, and low-density lipoprotein were incorporated into the model (**Table 5**). Results showed that advanced CKM stages remained higher risk of progressing to ESRD compared to CKM stage 2a (Stage 2b: SHR=2.737, 95% CI= 1.149–6.518; Stage 3: SHR=1.805, 95% CI=0.713–4.565; Stage 4: SHR=1.578, 95% CI=0.608–4.095). Notably, stage 2b patients exhibited a statistically significant increase in ESRD risk (p=0.023) (**Figure 4**). These findings align with the multivariate COX regression analysis, confirming the robustness of the results.

**Table 5:** Analysis of risk factors associated with end-stage renal disease (ESRD) in CKD patients, accounted for the competing risk of all-cause mortality when analyzing progression to ESRD (Fine-Gray sub-distribution hazard model).

Items	SHR (95%CI)	p value
CKM stage		
2a	reference	
2b	2.737 (1.149-6.518)	0.023
3	1.805 (0.713-4.565)	0.212
4	1.578 (0.608-4.095)	0.349
Cause of CKD		
glomerular nephritis	reference	
Hypertensive nephropathy	1.022 (0.613-1.703)	0.933
diabetic nephropathy	1.003 (0.558-1.801)	0.993
polycystic kidney	2.811 (1.176-6.719)	0.020
Other causes	1.159 (0.704-1.911)	0.562
eGFR (ml/min/1.73m2)		
<30	4.691 (2.232-9.862)	<0.001
30-60	1.324 (0.727-2.411)	0.359
>60	reference	
Hb (g/L)		
<110	1.664 (1.093-2.535)	0.018
>110	reference	
Albumin (g/L)		
<30	1.355 (0.839-2.187)	0.214
>30	reference	
Ca*P (mg2/dl2)		
<30	1.0850.765-1.540)	0.647
30-40	reference	
>40	1.274 (0.843-1.925)	0.250
LDL-C (mmol/L)		
<1.8	reference	
1.8-2.6	0.554 (0.308-0.995)	0.048
>2.6	0.791 (0.446-1.403)	0.422

CKD, chronic kidney disease; CKM, cardiovascular-kidney-metabolic syndrome; Hb, hemoglobin; LDL-C, Low-Density Lipoprotein Cholesterol; eGFR, estimated glomerular filtration rate.

## 4 Discussions

Our study results indicate that the presence of high-risk CKM increases the risk of adverse clinical outcomes in CKD patients, including all-cause mortality and progression to ESRD. Additionally, anemia, reduced eGFR, hypoalbuminemia, and the primary disease type of CKD are also risk factors for adverse clinical outcomes in CKD patients.

Li et al. compared the risk of all-cause mortality among patients with different CKM stages through a large-sample Chinese population study cohort (3). The study found that compared with the population with CKM risk stage 0, the cumulative risk of all-cause mortality over time increased with the increase in CKM stage, and this was more pronounced in the population diagnosed with CKM stage 3 and 4. This is similar to our research results. For the subgroup population that already has CKD, having a higher stage of CKM syndrome is still a risk factor for all-cause mortality, which is consistent with the non-CKD population. But it should be noted that although the p-value is close to the traditional significance threshold, the confidence interval of the OR in CKM stage 3 and 4 is wide (stage 3: 0.988–16.320; stage 4: 1.329–24.698), and the effect may range from slightly harmful to beneficial, which still requires further research for validation.

Type 2 diabetes (T2D), chronic kidney disease (CKD), atherosclerotic cardiovascular disease (ASCVD), and heart failure (HF) share overlapping etiologies and risk factors, and these conditions often coexist in the same patient. Medications used to control the risk factors of T2D, ASCVD, or CKD can also extend beyond their primary indications, providing benefits across a range of diseases (2). Therefore, the Cardiovascular-Kidney-Metabolic Syndrome (CKM) has been proposed to define the interactions among these diseases, emphasizing the interdisciplinary management of these conditions (1). CKD is a crucial component of CKM, and its stage should be considered in disease assessment of CKD. However, as a new concept, the evaluation methods and stage of CKM syndrome still require ongoing refinement.

Through multivariate Cox regression analysis, our results indicate that a high CKM stage has an independent predictive role for adverse clinical outcomes in CKD patients. As the CKM stage increases, the risk of all-cause mortality in CKD patients gradually rises. We also conducted analyses using the Fine-Gray subdistribution model to assess risk factors for progression to ESRD in CKD patients while accounting for competing risks from all-cause mortality. The results demonstrated consistency with those obtained from the Cox regression model, confirming their alignment with real-world clinical scenarios. However, in the risk prediction model for progression to ESRD in CKD patients, there was no significant difference between higher CKM risk levels. This may be because most patients with higher CKM risk levels experience cardiovascular accidents or other life-threatening adverse events before they need to start renal replacement therapy, leading to death without the opportunity to progress to ESRD requiring renal replacement therapy. In assessing the risk of ESRD in patients, we should also pay attention to other affections, including the etiology of CKD (polycystic kidney), anemia and hypoalbuminemia.

Currently, there is no unified standard for the risk stratification of CKM syndrome. In the CKM risk stage recommendations proposed by the AHA, CKM syndrome is divided into 5 stages, from low to high: no related risk factors, potential metabolic risk, presence of metabolic diseases, subclinical organ damage, and clinical CVD lesions. Among these, stage 2 is defined as the combination of metabolic diseases or kidney disease related to the heart, including visceral fat accumulation, diabetes, hypertension, hyperlipidemia, overweight, and CKD (1). Our research mainly adopted the CKM stratification method recommended by AHA. Additionally, because our study subjects are CKD population, we further divided CKM stage 2 into patients with simple comorbid CKD (2a) and patients with both CKD and other metabolic risks (2b), in order to compare the impact of additional metabolic risks on CKD patients (**Table 1**). Our study indicates that CKD patients with metabolic diseases (CKM risk stage 2b) have a further increased risk of all-cause mortality or progression to ESRD compared to those without other metabolic diseases (CKM risk stage 2a), and this is the most significant risk factor for the occurrence of ESRD in patients. This suggests that there is still gap for refinement in the CKM risk stage method. For instance, when evaluating a patient’s cardiorenal metabolic risk, it is important to consider the cumulative calculation of metabolic risk factors with CKD. Diabetes is a major cause of CKD, and persistent hyperglycemia activates inflammatory responses, leading to glomerular and tubular damage (7, 8). Elevated blood glucose alters the structure of lipoproteins (8), increasing the risk of lipid metabolism disorders and other metabolic diseases, as well as the risk of cardiovascular events. CKD patients with diabetes have higher coronary artery calcium (CAC) scores and a higher incidence of adverse cardiovascular events compared to the non-diabetic population (9). Hypertension has a bidirectional relationship with CKD, and elevated blood pressure is associated with poor renal prognosis (10).

In the high-risk stages of CKM syndrome (stage 3 or 4), potential or clinical stage cardiac and renal function impairment is the primary grading criterion (1). Cardiac function is closely related to changes in renal function. In our study population, cardiovascular disease were the leading cause of all-cause mortality, accounting for 39.8% of all deaths. This is consistent with the epidemiological statistics of the East Asian population (11). According to the epidemiological survey from the GBD database, renal insufficiency and other metabolic risks are risk factors for the occurrence of stroke, coronary heart disease, and other cardiovascular and cerebrovascular diseases in CKD patients (12). Reduced kidney function is positively correlated with the occurrence and mortality risk of coronary atherosclerotic heart disease (13). Even a mild decline in eGFR can reshape the cardiac hemodynamic structure through oxidative stress reactions, leading to changes in cardiac function and perfusion (14). Conversely, cardiac dysfunction also affects renal function. The eGFR of patients with chronic heart failure tends to decline continuously, and they are more prone to renal failure compared to those with healthy cardiac function. The rate of eGFR decline is more pronounced in patients with diabetes and lower baseline eGFR (15).

In our study, both higher and lower levels of low-density lipoprotein cholesterol (LDL-C) in CKD patients were associated with an increased risk of progression to ESRD. However, lipid levels did not have significant predictive value for mortality risk in CKD patients. Low LDL-C levels may indicate malnutrition in patients, which is considered one of the risk factors for adverse outcomes in CKD patients (4). Similar to the non-CKD population, elevated LDL-C is a risk factor for atherosclerotic events in CKD patients (16). The use of statins and other medications to lower LDL-C is an effective means of reducing the incidence of ASCVD events, but its benefits for CKD patients are controversial. Studies on non-dialysis CKD populations have shown that the use of statins or other LDL-C-lowering drugs does not significantly reduce all-cause mortality (17, 18). However, in some studies, lowering LDL-C can still reduce the risk of cardiovascular events in CKD patients (16) and slow the progression of kidney damage (19). For CKD patients, it is still necessary to pay attention to and control LDL-C levels (20).

Our study still has some limitations. Currently, there are no universally accepted imaging diagnostic criteria for subclinical heart failure and subclinical atherosclerotic cardiovascular disease used in CKM stage 3 (21). Subclinical heart failure typically refers to patients who exhibit abnormalities in imaging or related serum markers but do not show clinical symptoms, which includes patients with normal levels of pro-B-type natriuretic peptide (NPpro-BNP) who have already experienced cardiac structural changes (left ventricular hypertrophy, chamber dilation, wall motion abnormalities, valvular or myocardial tissue abnormalities) or functional abnormalities (reduced left or right ventricular systolic function or diastolic dysfunction), or those with abnormal serum markers but no cardiac structural or functional abnormalities (22). In patients not diagnosed with heart failure, mild elevations in NPpro-BNP alone (23) or left ventricular structural and functional abnormalities (24) can also increase the risk of adverse outcomes. Combining imaging examinations with serum marker tests can provide a more comprehensive assessment of the patient’s cardiac function. Additionally, our results were limited by the sample size and the innate shortcomings of an observational study, which has led to potential inaccuracies in some results, including the wider confidence intervals for CKM3 and 4 when calculating the risk of mortality. A larger prospective study is needed to further solidify our findings.

Overall, our center’s research findings are similar to the existing conclusions regarding the association between different stages of CKM syndrome and mortality risk (24). Additionally, our study results also demonstrate the impact of high-risk CKM on renal function decline in the CKD population.

## 5 Conclusion

A high-risk CKM stage can predict adverse clinical outcomes in CKD patients. In the long-term management of CKD patients, it is important to focus on the risk management of cardiorenal metabolic diseases, especially early screening and control of metabolic comorbidities and subclinical cardiovascular diseases, which can positively impact the prognosis of CKD patients. Additionally, attention should be given to the potential effects of the patient’s primary disease type and other comorbidities on the progression of CKD.

## Data Availability

The raw data supporting the conclusions of this article will be made available by the authors, without undue reservation.
